# The Temporal Profiles of Changes in Nerve Excitability Indices in Familial Amyloid Polyneuropathy

**DOI:** 10.1371/journal.pone.0141935

**Published:** 2015-11-03

**Authors:** Hsing-Jung Lai, Ya-Wen Chiang, Chih-Chao Yang, Sung-Tsang Hsieh, Chi-Chao Chao, Ming-Jen Lee, Chung-Chin Kuo

**Affiliations:** 1 Department of Neurology, National Taiwan University Hospital, Taipei, Taiwan; 2 Institute of Anatomy and Cell Biology, National Taiwan University College of Medicine, Taipei, Taiwan; 3 Department of Medical Genetics, National Taiwan University Hospital, Taipei, Taiwan; 4 Institute of Physiology, National Taiwan University College of Medicine, Taipei, Taiwan; University of Sydney, AUSTRALIA

## Abstract

Familial amyloid polyneuropathy (FAP) caused by a mutation in transthyretin (*TTR*) gene is an autosomal dominant inherited disorder. The aim of this study is to explore the pathophysiological mechanism of FAP. We prospectively recruited 12 pauci-symptomatic carriers, 18 patients who harbor a *TTR* mutation, p.A97S, and two-age matched control groups. Data of nerve excitability test (NET) from ulnar motor and sensory axons were collected.NET study of ulnar motor axons of patients shows increased threshold and rheobase, reduced threshold elevation during hyperpolarizing threshold electrotonus (TE), and increased refractoriness. In sensory nerve studies, there are increased threshold reduction in depolarizing TE, lower slope of recovery and delayed time to overshoot after hyperpolarizing TE, increased refractoriness and superexcitability in recovery cycle. NET profiles obtained from the ulnar nerve of carriers show the increase of threshold and rheobase, whereas no significant threshold changes in hyperpolarizing TE and superexcitability. The regression models demonstrate that the increase of refractoriness and prolonged relative refractory period are correlated to the disease progression from carriers to patients. The marked increase of refractoriness at short-width stimulus suggests a defect in sodium current which may represent an early, pre-symptomatic pathophysiological change in TTR-FAP. Focal disruption of basal lamina and myelin may further increase the internodal capacity, manifested by the lower slope of recovery and delayed time to overshoot after hyperpolarization TE as well as the increase of superexcitability. NET could therefore make a pragmatic tool for monitoring disease progress from the very early stage of TTR-FAP.

## Introduction

Familial amyloid polyneuropathy (FAP) caused by mutations of transthyretin (TTR) is the most common cause of FAP, which is a multisystem disorder with autosomal dominant transmission [1]. Patients with TTR-FAP frequently present with a rapidly progressive distally symmetric sensorimotor polyneuropathy, cardiac dysfunction associated with autonomic failure, and a fatal outcome [2, 3]. The symptoms of neuropathy usually begin from distal lower extremities with prickling, tingling and burning sensations indicating involvement of small nerve fibers [4, 5]. The paresthesias may progress relentlessly from feet up to legs, and impairment of light touch and deep sensation with motor deficits would usually ensue. With the loss of large fiber sensation, the patient would have more difficulties in walking and balance. Motor deficits also follow a length-dependent pattern, and thus are usually characterized by marked muscle wasting in lower limbs and hands [4, 6].

TTR protein is encoded by the *TTR* gene which is a transporter for serum thyroxine and retinol binding proteins [7, 8]. Currently, there are more than 110 causative *TTR* mutations in the four coding exons (http://www.hgmd.cf.ac.uk/ac/all.php). Among them, the missense p.V30M mutation is probably the most prevalent one across the world [2, 6, 8]. The p.A97S mutation is the most common one among the patients in Taiwan. The age of onset for patients with p.A97S TTR-FAP is usually in their seventh decade of life, later than those with the p.V30M mutation [9]. Moreover, in contrast to those with p.V30M mutation, neuropathy in p.A97S TTR-FAP patients affects both large- and small- fibers to a similar degree [9].

Albeit conventional nerve conduction and pathological studies have been extensively documented [10–12]. These data are chiefly from symptomatic patients or relatively late stage of the disease. Detailed functional characterizations, especially longitudinal studies from the pre-symptomatic to the symptomatic stages, are lacking. Nerve excitability test (NET) is an electrophysiological tool, using threshold tracking technique to evaluate detailed physiological functions of sensory and motor axons [13, 14]. Since the symptoms of p.A97S TTR-FAP usually begin at the seventh decade of life and the genetic diagnosis is readily available, it is feasible to recruit a group of asymptomatic carriers for early investigation and longitudinal follow-up, so that the temporal profiles of abnormalities in clinical and electrophysiological parameters can be established. We therefore employed NET to make a comparative study between asymptomatic p.A97S TTR-FAP carriers and symptomatic patients, to explore the functional and relevant pathophysiologic changes associated with disease progress.

## Material and Methods

### Subjects

Patients with TTR-FAP were recruited from the Department of Neurology, National Taiwan University Hospital, Taipei, Taiwan. The family members who carry the *TTR* mutation p.A97S were also recruited for the study. For each subject, the information of medical history, neurologic examinations, neurological disability score (NDS, range 0–172), overall neuropathy limitation scales (ONLS, range 0–12) and conventional nerve conduction study were collected [15, 16]. The carriers are either asymptomatic or have only equivocal subjective sensory symptoms (pauci-symptomatic carriers). The pauci-symptomatic carrier has a NDS below 10 and no evidence of polyneuropathy in nerve conduction study. Because of the age disparity between patient- and carrier-groups, two age-matched control groups (NC1 for the carriers and NC2, patients) were recruited. All of the subjects provided their written informed consent, and all procedures were approved by the Research Ethics Committee of the National Taiwan University Hospital (201302064RINC), Taipei, Taiwan.

### Nerve excitability test

Nerve excitability test (NET) was carried out to evaluate the electrophysiological properties of motor and sensory axons in both patients and carriers. Considering that carpal tunnel syndrome is frequently found in patients with TTR-FAP and may confound the result of electrophysiologic test, we chose to study the motor and sensory axons from the ulnar nerve. The stimulation site for the left ulnar nerve was proximal to the wrist crease, and the recording site (for compound motor action potential, CMAP) was at the first dorsal interosseous muscle. The pick-up site for sensory nerve action potential (SNAP) was at the lateral aspect of the left fifth metacarpophalangeal joint. The NET test was carried out following the TRONDNF protocol (Version 18/8/2008, copyright, Prof. Hugh Bostock, Institute of Neurology, London) [13, 14]. The currents required to elicit a potential (CMAP or SNAP) to attain 40% of maximal response level were tracked. Skin temperature was kept above 34°C.

Each protocol cycled through five subroutines including 1) stimulus response curve, 2) strength duration relationship, 3) recovery cycle, 4) threshold electrotonus with 20% and 40% subthreshold depolarization and hyperpolarization, and 5) current-threshold (I/V) relationship. The first subroutine generated a stimulus-response curve with the triggering stimuli set at 1 ms for motor axons and 0.5 ms for sensory axons. For the stimulus response curves, a gradual increase of stimulus current up to a supramaximal level was delivered to obtain the peak response, and the stimulus for 50% depolarization was shown. In the second subroutine, the stimulus duration was gradually reduced from 1 to 0.2 ms (0.5–0.1 ms for sensory axons) to depict the strength duration curve. The strength-duration time constant (SDTC,τ_SD_) was calculated using the Weiss’ equation. The recovery cycle is characterized by changes in axonal excitability following a supramaximal conditioning stimulus. The cycle includes a relative refractory period (at short inter-stimulus intervals), superexcitable period (when the threshold is reduced), and subexcitable period (when the nerve is less excitable). The changes in threshold current were recorded at the end of each of the eighteen conditioning test intervals from 2 to 200 ms. The fourth and fifth subroutines measure the capacity of prolonged subthreshold currents to alter the potential difference across the nodal and internodal membranes. The change in threshold due to the electrotonic changes in membrane potential of a sampled axon is defined as the threshold electrotonus (TE). Different durations (up to 100 ms) of the preconditioning eletrotonus current sets at ±40% and ±20% (TE_d_ for depolarization and TE_h_ for hyperpolarization) of the threshold level were applied, and a test pulse was delivered during or after the preconditioning current. The TE plot recorded the reduction in threshold at different time intervals. The current-threshold relationship (similar to the current-voltage [I/V] relationship) is defined as the change in threshold voltage following a 200-ms subthreshold polarizing current. From +50% (depolarizing) to –100% (hyperpolarizing) of the threshold current, the strength of the applied polarizing conditioning current was altered in 10% decrement. The current-threshold relationship is defined as the current plotted against the threshold reduction. To obtain the refractoriness in recovery cycle and to detect the threshold after hyperpolarizing current in current threshold (I/V) relationship, the threshold could be quite high. For safety reasons, the upper limit of stimulus current was set at 50 mA, and the test would be stopped if this limit was reached. The data of the unfinished set would not be enrolled for analysis.

### Data analysis

The software QTRACP (copyright, Prof. Hugh Bostock, Institute of Neurology, London) and SPSS version 17 (SPSS, Chicago, IL) were employed for data analysis. Tukey’s outlier filter method was used to exclude the outliers. The observation Y was considered as an outlier, if Y < (Q1 − 1.5 IQR) or Y > (Q3 + 1.5 IQR), where Q1 = lower quartile, Q3 = upper quartile, and IQR = (Q3 − Q1) is the interquartile range. An outlier out of seven observations in hyperpolarizing TE of sensory axons, which has extreme fanning out, was thus removed from analysis. Mann-Whitney U-test were used for non-parametric comparison among patient, carrier and control groups. For correlation between excitability indices and disease severity, stepwise linear regression models were applied. Despite that there is variable penetrance and age of onset across the patients with different mutations, the penetrance in general increases with age [17, 18]. Thus, for evaluation of the temporal changes from preclinical to clinical states, linear or polynomial functions were used to fit the age-related changes in carriers and patients. The model with the highest R^2^ value was reported. *P* values < 0.05 were regarded as statistically significant. In each figure, the error bars indicate the standard error of mean (S.E.M.).

### Computer modeling

A mathematic model for human myelinated axon developed in previous studies was adopted to simulate the NET findings from patients [19–21]. The modelling was performed with the MEMFIT program [22]. The discrepancy between simulated model and tested excitability findings was calculated by the weighted sum of the squares of the error terms: [(x_m_-x_n_)/s_n_]^2^, where x_m_ is the simulated threshold from the computer modeling, x_n_ is the mean value from the data of NET test and s_n_ is the standard deviation of the values. The weighting of model optimization was the same for all thresholds of the same type and were chose to be equal to all subroutines, including threshold-charge relationship, threshold electrotonus, I/V relationship and recovery cycle [23]. The model was optimized by an iterative least squares procedure, so that the discrepancy is minimized.

## Results

### Clinical profiles

Eighteen patients (14 male and 4 female) and 12 carriers (8 male and 4 female) with p.A97S *TTR* mutation were enrolled in the study. The clinical demographic data for both patients and carriers are listed in Table 1. The mean age of patients (65.2±5.4 years) is significantly older than carriers (39.4±8.3 years, p<0.001). Two age-matched groups of controls are NC1 (38.4±11.2 years) for carriers and NC2 (58.6±14.7 years) for patients, with 30 subjects in each group. The mean disease duration is 2.9±1.4 years (range, 0.6–6 years). The median neurological disability score (NDS) and overall neuropathy limitation score (ONLS) for the patients are 54.5 (13–82) and 4.5 (3–9), respectively. The nerve conduction studies in carriers are unremarkable. Reduced CMAPs and SNAPs with mild slowing of nerve conduction velocities are found in the ulnar and peroneal nerves of the patients (Table 1). SNAP is either absent or too small for stable threshold tracking in 11 of the patients, in whom nerve excitability test for ulnar sensory axon was not performed.

**Table 1:** Demographic data of carriers and patients with familial amyloid polyneuropathy.

	Carrier (N = 12)	Patient (N = 18)	Reference range
Age	39.4±8.3 (29–60)	65.2±5.4 (58–75)	
Gender (male)	8	14	
Disease duration	NA	2.9±1.4	
Muscle power (MRC scale)			
UE proximal*	5	4 (3–5)	
UE distal*	5	4(3–5)	
LE proximal*	5	4(3–5)	
LE distal*	5	3.5(0–5)	
NDS*	0 (0–8)	54.5 (13–82)	
ONLS*	0 (0–1)	4.5 (3–9)	
Ulnar nerve CMAP (mV)	18.9±2.2	4.08±2.35	>6
Ulnar MNCV (m/s)	61.1±4.4	51±4.75	>50
Ulnar SAP (microV)	40.3±13.7	3.61±3.55	>8.5
Ulnar SNCV (m/s)	59.3±8.1	45.3±3.69	>50
Peroneal CMAP (mV)	6.00±1.97	0.46±0.68	>2
Peroneal MNCV (m/s)	49.3±4.0	39.7±5.6	>40
Sural SAP	18.6±8.1	0.78±1.35	>5
Sural SNCV	49.6±4.6	38.6±3.6	>40

Data are shown in Mean ± S.D. unless mentioned.

*: median (range)

The reference range is the standard range in National Taiwan University Hospital. MRC: Medical research council; UE: upper extremities; LE: lower extremities; CMAP: compound motor action potential; MNCV: motor nerve conduction velocity; NDS: Neurological Disability Score; SAP: sensory action potential; SNCV: sensory nerve conduction velocity; ONLS: overall neuropathy limitation score.

### Stimulus response and strength-duration properties

**Fig 1:**
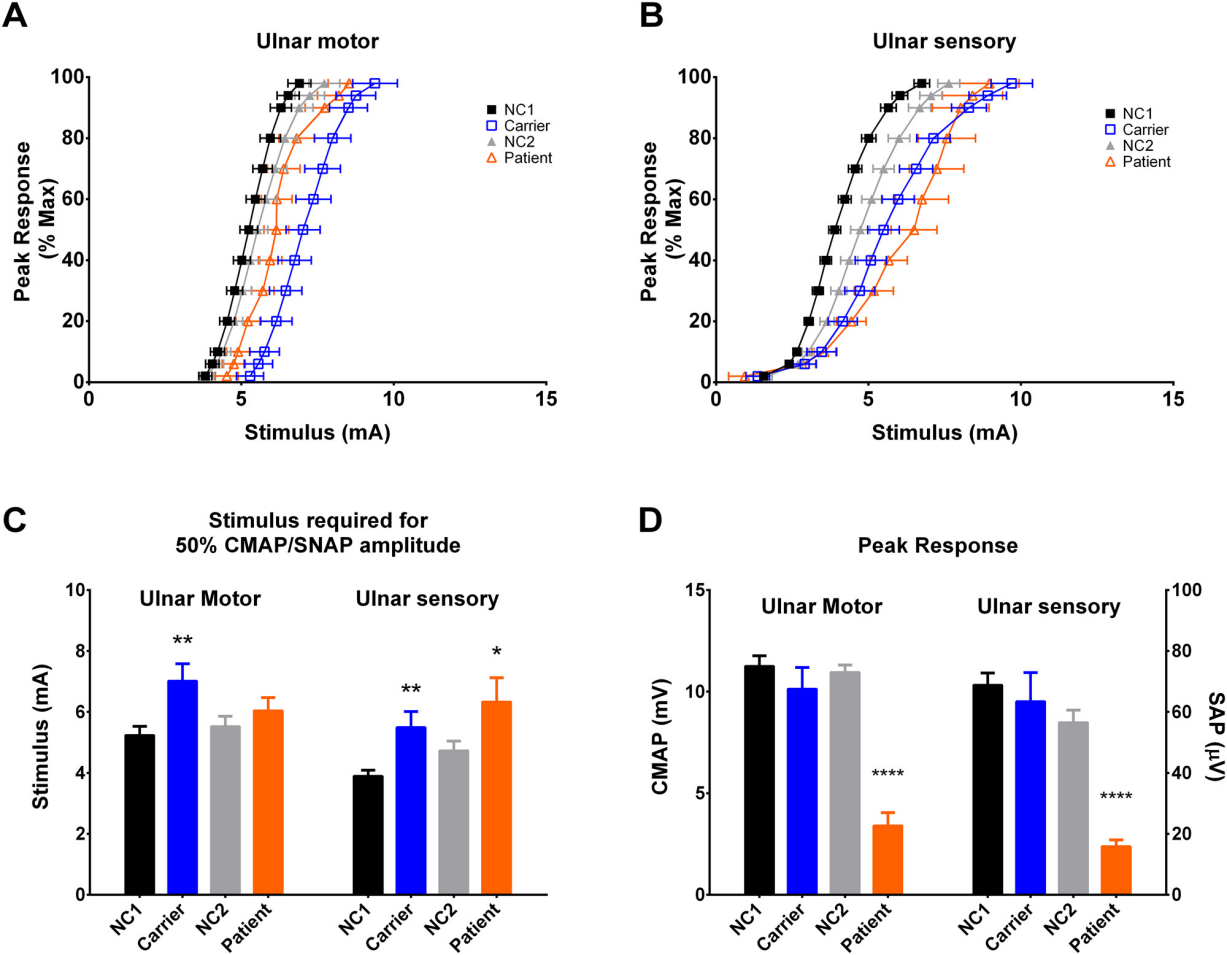
Stimulus-response properties of the ulnar nerve. **A and B**, Stimulus-response curves from the ulnar nerve of carriers (blue open squares), patients (orange open triangles) and two groups of age-matched controls, NC1 (black filled squares) for carriers and NC2 (gray filled triangles) for patients, are shown [The lines connecting the data points are drawn by hand]. The stimulant current to obtain 50% of CMAP (stimulus for 50% depolarization) in carriers was significantly higher for carriers (7.02±0.56 mA) than for NC1 (5.24±0.29 mA) (*p* = 0.0096). **C**, The motor threshold in patients was not significantly increased, as compared to NC2 (6.05±0.43 mA for patients v.s 5.53±0.33 mA for NC2, *p* = 0.33). In sensory axons, the stimulus for 50% depolarization was significantly increased in both carriers (5.5±0.52 mA for carriers v.s. 3.9±0.19 mA for NC1, *p* = 0.0035) and patients (6.34±0.79 mA for patients v.s. 4.74±0.31 mA for NC2, *p* = 0.04). **D**, There are no significant differences in peak responses of both CMAP and SNAP between carriers and NC1. However, the CMAP (3.4±0.6 mV) and SNAP (15.9±2.2 μV) for patients are significantly smaller than that of NC2 (11±0.4 mV and 56.6±4 μV, both *p* < 0.0001). *: *p*<0.05, **: *p* <0.01, ***: *p* <0.001, ****: *p* <0.0001.

(Fig 1A and 1B) shows the stimulus response curves of both motor and sensory axons of the ulnar nerve from patients, carriers and controls. Compared to the age-matched controls, the threshold of motor and sensory axons is evidently higher in patients and carriers. The stimulus currents to achieve 50% of peak CMAP or SNAP in motor and sensory axons are significantly higher in carriers than in NC1 and in patients than in NC2 (Fig 1C). The stimulus for 50% depolarization for motor axons between patients and NC2 does not show a significant difference, a finding probably ascribable to the relatively increased threshold in older normal subjects (Fig 1D). These findings indicate an early involvement of both motor and sensory axons even in asymptomatic carriers with TTR-FAP.

**Fig 2:**
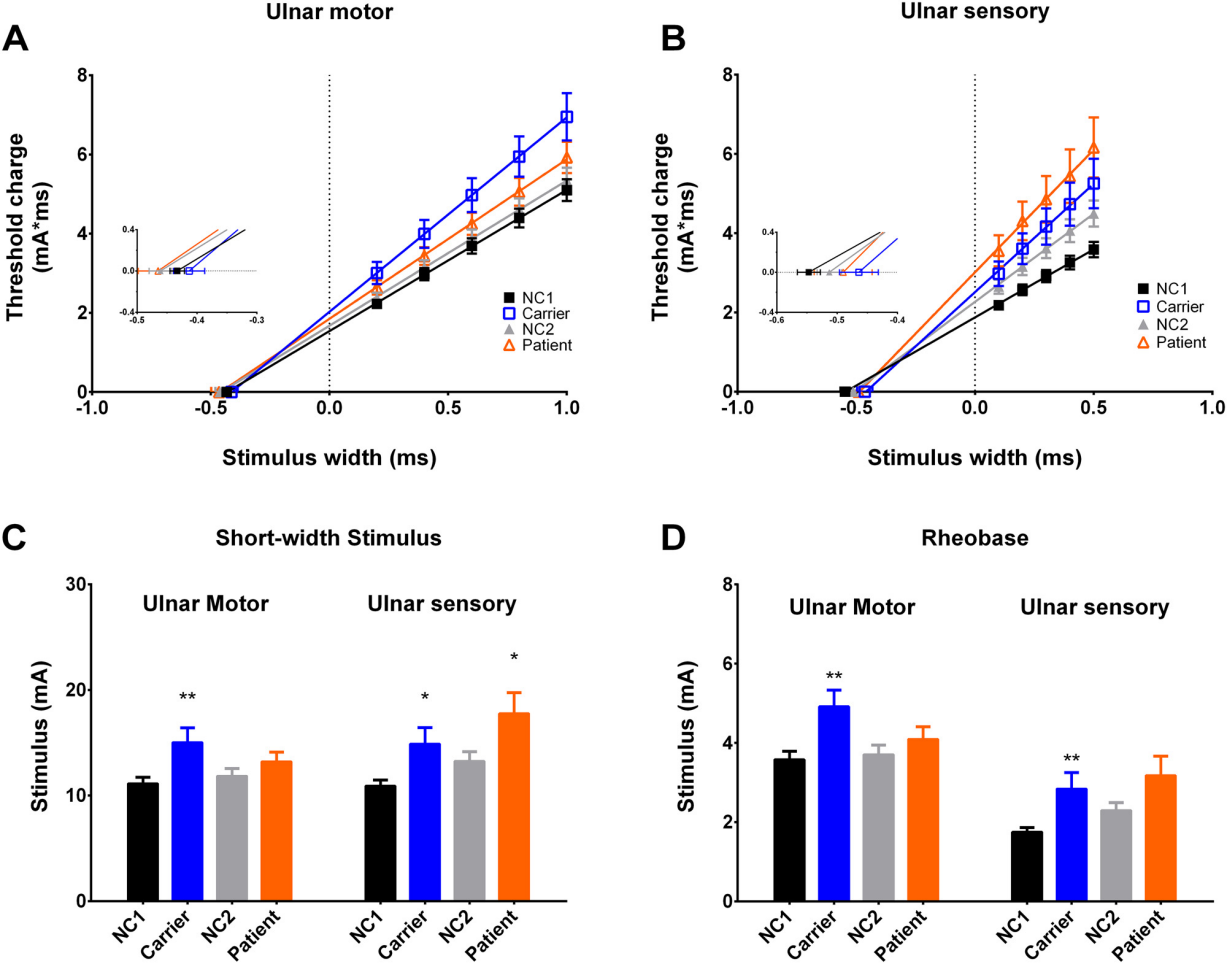
Strength-duration properties of the ulnar nerve. **A and B**, The threshold charge-stimulus width curves for motor and sensory axons are shown (the meaning of the symbols are the same as in Fig 1). The slope of the line represents the rheobase and the absolute value of x-intercept is equivalent to the strength duration time constant (SDTC). The lines are linear regression fits to the data points. In sensory axons, the SDTC is significantly decreased in carriers (0.46±0.03 ms for carriers v.s. 0.55±0.02 ms for NC1, *p* = 0.03), but no significant difference between patients (0.49±0.05 ms) and NC2 (0.51±0.02 ms, *p* = 0.39). On the other hand, there is no significant change of SDTC in motor axons. **C and D**, In ulnar motor axons of carriers, the threshold in very short duration (0.2 ms) (15.02±1.39 mA for carriers vs. 11.13±0.60 mA for NC1, p = 0.015) and the rheobase (4.92±0.42 mA for carriers vs. 3.58±0.21 mA for NC1, *p* = 0.0082) are significantly increased. There is no significant change in both threshold in short stimulus width (0.2 ms) and rheobase in motor axons of patients. In sensory of axons of carriers, the increases of both threshold in short stimulus width (0.1ms) (17.76±1.98 mA for carriers vs. 13.26±0.89 mA for NC1, *p* = 0.025) and rheobase (2.84±0.41mA for carriers vs. 1.75±0.11 mA for NC1, *p* = 0.0058) are significant. *: *p*<0.05, **: *p* <0.01.

The strength-duration time constants (SDTC) of carriers and patients in motor axons are not significantly different from that of controls (Fig 2A). For sensory axons, SDTC for carriers is significantly reduced as compared to NC1 (Fig 2B). However, the difference of SDTC of sensory axons between patients and NC2 does not reach a significant level. Compared to NC1, the threshold currents at short-width stimulus (0.2 ms for motor and 0.1 ms for sensory studies) as well as the rheobase of both motor and sensory axons are significantly increased in carriers (Fig 2C and 2D). Although the stimulus currents for short-width stimulus in sensory axons are also significantly larger than that in NC2, there is no difference in stimulus currents for the threshold and rheobase in motor axons. In summary, the increase of the stimulus current to attain 40% of peak response with short-width stimulus strongly implicates a defective nodal property, especially the transient sodium or slow potassium currents (see below and Discussion).

### Recovery cycle

**Fig 3:**
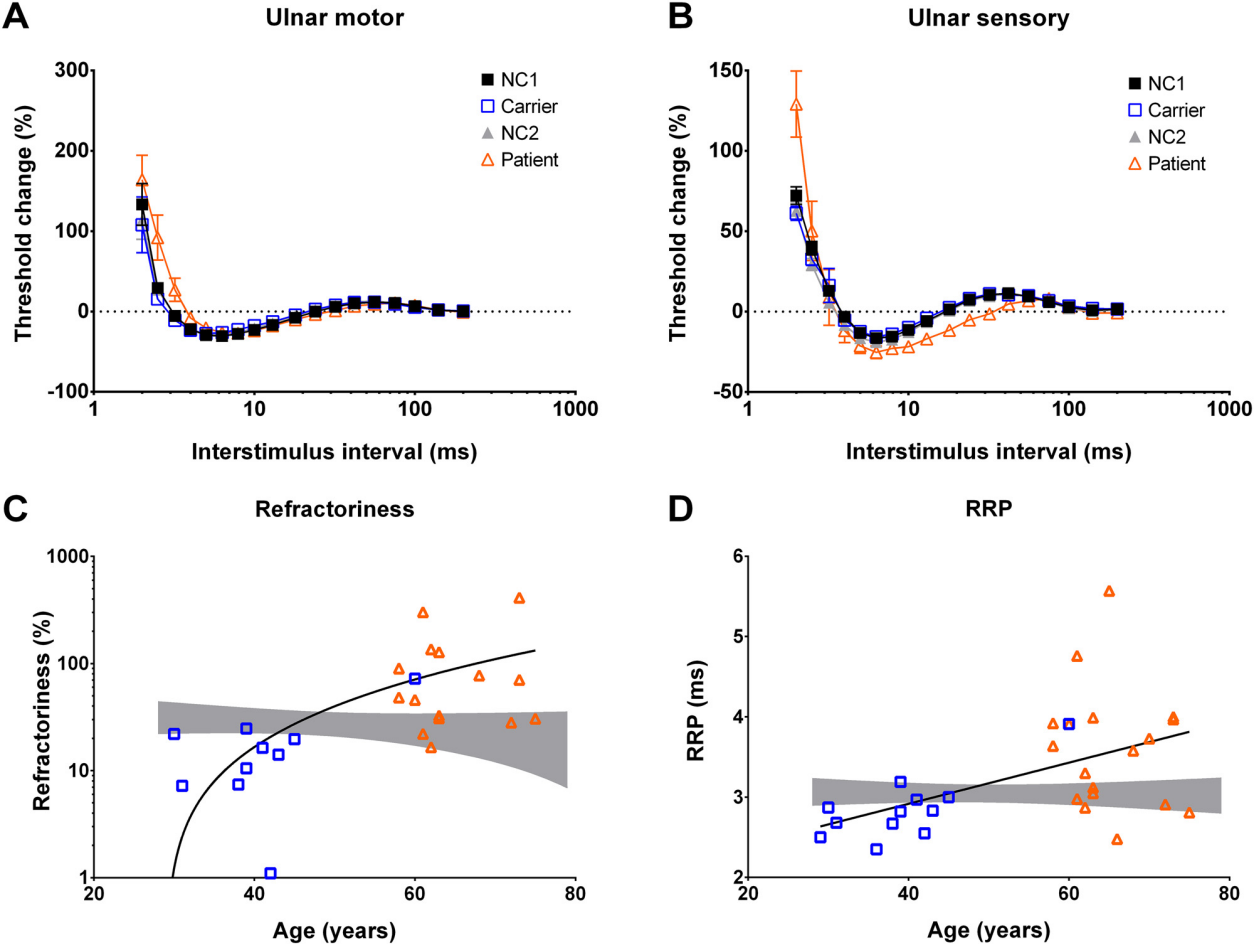
Recovery cycle analysis and regression models demonstrating the refractoriness and relatively refractory period (RRP) against age. **A**, Compared to NC1 and NC2 (the meaning of symbols are the same as in Fig 1), the refractoriness (at 2.5 ms, 91.8±28.0% for patient vs. 27.1±4.5% for NC2, *p* = 0.007) and relatively refractory period (RRP) (3.59±0.18% for patient vs. 3.04±0.07% for NC2, *p* = 0.0083) are significantly elevated in motor axons of patients, but not in carriers. The late subexcitability is also decreased in patients (9.9±0.6% for patients vs. 12.3±0.9% for NC2). [The lines connecting the data points are drawn by hand.] **B**, In the sensory axons, the refractoriness in patients is not markedly increased until interstimulus interval was 2 ms (129.2±20.6% for patients vs. 62.6±5.6% for NC2, p<0.01). The superexcitability is also significantly increased in patients (-25.7±2.6% for patients vs. -18.2±1% NC1, *p* = 0.011) [The lines are drawn by connecting the mean value of each spot.]. **C and D**, The refractoriness and RRP are increased by age and the evolution of disease (from carrier to patients). The gray band is the 95% confidence interval for controls. The quadratic regression model (Y = 72.04+5.03*X+0.082*X^2^) fits the data with a R^2^ value (0.2159), demonstrating the correlation between the refractoriness and progress (severity) of the disease. On the other hand, the correlation between RRP and progress of disease is fitted by the linear regression model (Y = 0.02563*X + 1.892) with a R^2^ value of 0.257.

If there are sodium channel dysfunctions at the nodal membrane, then the percentage of refractoriness, subexcitability and the time for relatively refractory period (RRP) in the recovery cycle test would also tend to be altered. As shown in Fig 3A and Table 2, the percentage of refractoriness at 2.5 ms is significant higher in motor axons of patients than that of NC2 (Table 2). The RRP of the motor axons of patients is also markedly prolonged (Table 2). Compared to NC2, the percentage of subexcitability is significantly reduced in patients (Table 2). However, there is no significant difference between carriers and NC1. To test whether progress of the disease has an influence on these parameters, the regression models with the highest R^2^ value for refractoriness and RRP related to aging were analyzed (Fig 3C and 3D). There seems to be a trend of decreasing refractoriness with normal aging (gray band in Fig 3C). In sharp contrast, the refractoriness increases along with age from carriers to patients (Fig 3C). Likewise, the RRP is prolonged as the disease progresses, but it tends to keep constant or even shows a slight trend of decrease in control (gray band in Fig 3D). In sensory axons, there is no significant increase of refractoriness percentage at an interstimulus interval of 2.5 ms, but the percentage is markedly raised with shorter intervals. There is also an evidently enhanced superexcitability in patients (Fig 3B). These findings consistently indicate that nodal dysfunction characterized by elevated threshold, increment of refractoriness, and prolonged RRP, is the primary functional change in TTR-FAP. These primary functional changes, together with the reduced superexcitability and subexcitability in motor axon studies, provide strong evidences for the reduced transient sodium currents in TTR-FAP. The reduced subexcitability, on the other hand, would argue against the significantly increased nodal slow potassium currents (see Discussion for details). The increased superexcitability and the high threshold in sensory axons, nevertheless, may suggest the coexistence of the other membrane abnormalities, such as membrane hyperpolarization or altered internodal capacitance (see below).

**Table 2:** Comparison of nerve excitability properties of ulnar nerves among controls, carriers and patients with familial amyloid polyneuropathy.

	NC1	Carrier	NC1 to carrier P value	NC2	patient	NC2 to patient P value
Motor axon						
Latency	6.59±0.07	6.53±0.21	0.24	6.64±0.08	8.42±0.32	<0.0001
Threshold Electrotonus						
TEd_(10-20ms)_	65.7±0.7	63.3±0.9	0.037	66.6±0.9	63.9±1.2	0.069
TEd_(90-100ms)_	45.5±0.6	42.6±1.4	0.07	45.5±0.9	44±1.5	0.31
TEh_(10-20ms)_	-69.8±0.8	-68.6±1.3	0.27	-71.6±1.0	-65.2±2.0	0.0043
TEh_(90-100ms)_	-119.9±2.2	-124.3±4	0.23	-121±2.4	-115±6.1	0.27
TEh_(slope 101-140ms)_	1.72±0.04	1.86±0.07	0.039	1.78±0.04	1.56±0.1	0.049
I/V parameters						
Resting I/V slope	0.55±0.012	0.566±0.021	0.47	0.562±0.023	0.57±0.022	0.18
Hyperpolarizing I/V slope	0.33±0.013	0.346±0.026	0.58	0.352±0.012	0.451±0.039	0.012
Recovery cycle						
RRP (ms)	3.05±0.07	2.86±0.12	0.1063	3.04±0.07	3.59±0.18	0.0083
Refractoriness at 2.5ms (%)	29.2±5	15.6±5.8	0.089	27.1±4.5	91.8±28.0	0.007
Superexcitability (%)	-29.9±0.7	-26.3±2.1	0.33	-28.6±1.1	-26.7±2	0.233
Subexcitability (%)	11.3±0.8	10.9±2.1	0.47	12.3±0.9	9.9±0.6	0.029
Sensory axon						
Latency	2.91±0.04	2.86±0.1	0.61	2.99±0.05	3.91±0.26	0.0003
Threshold Electrotonus						
TE_d(10-20ms)_	60.3±0.9	57.6±1.9	0.44	60.5±0.8	75.3±4.1	0.0001
TE_d(90-100ms)_	48.5±1.2	46.9±1.8	0.7	50.7±1.3	47.7±4.1	0.49
TEh_(10-20ms)_	-79.1±1.6	-75.7±2.9	0.42	-83.2±1.6	-77.2±4.2	0.35
TEh_(90-100ms)_	-124.5±3.8	-126.3±4.9	0.8	-136.6±4.2	-132.5±11.1	0.93
TEh_(slope 101-140ms)_	2.21±0.06	2.31±0.12	0.45	2.41±0.06	1.87±0.13	0.002
Time to overshoot	33.9±1.8	36.5±2.0	0.419	37.2±1.9	49.0±5.0	0.024
I/V parameters						
Resting I/V slope	0.583±0.023	0.619±0.042	0.66	0.546±0.023	0.507±0.046	0.46
Hyperpolarizing I/V slope	0.303±0.011	0.316±0.018	0.46	0.347±0.015	0.446±0.03	0.02
Recovery cycle						
RRP (ms)	3.79±0.13	3.66±0.16	0.45	3.47±0.12	3.37±0.32	0.42
Refractoriness at 2.5ms (%)	39.4±4.2	32.6±4	0.37	29.3±3.7	50.2±18.5	0.33
Superexcitability (%)	-15.8±1	-14.8±1.3	0.74	-18.2±1	-25.7±2.6	0.011
Subexcitability (%)	10.9±0.6	12±1	0.44	10.6±0.6	6.9±1.9	0.063

Data are shown in Mean ± S.E.M

### Threshold Electrotonus (TE) and I/V relationship

**Fig 4:**
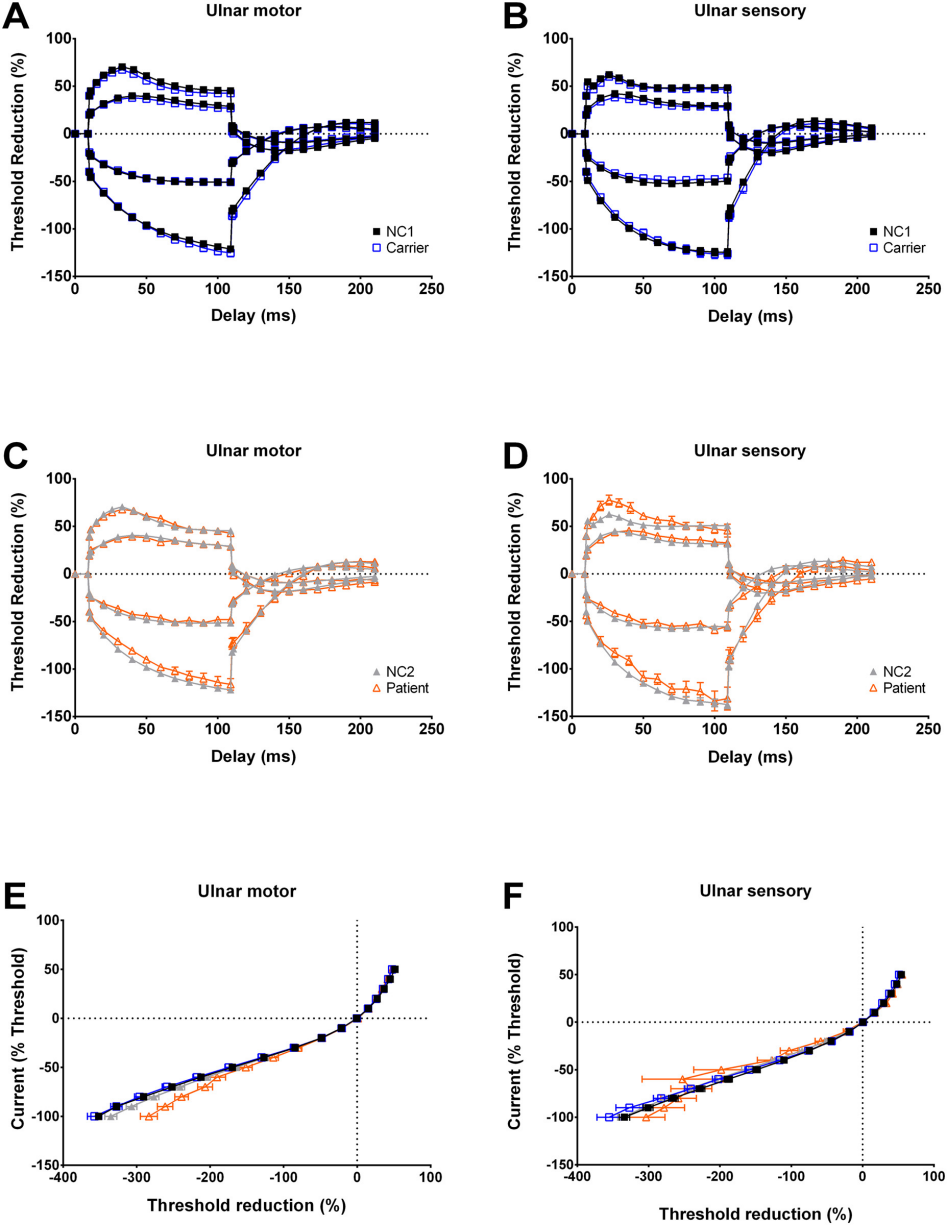
Threshold electrotonus (TE) and I/V relationship of the ulnar nerve. **A and C**, Compared to NC2, the threshold elevation in hyperpolarizing TE of motor axons is reduced in patients (TE_h(10-20ms)_ and TE_h(20-40ms)_, -65.2±2.0 mV and -80.4±2.6 mV for patients vs. -71.6±1.0 mV and -89.2±1.3 mV for NC2, *p* = 0.0043 and 0.0056, respectively). The recovery from hyperpolarization (TEh_(slope 101-140ms)_) is slightly less steep in patients (1.56±0.1 for patients vs. 1.78±0.04 for NC2, *p* = 0.049). However, it is slightly steeper in carriers (1.86±0.07 for carriers v.s. 1.72±0.04 for NC1, *p* = 0.039). In carriers, the threshold reduction is reduced in depolarizing TE (TE_d(10-20ms)_ and TE_d(90-100ms)_, 63.3±0.9 mV and 42.6±1.4 mV for carriers vs. 65.7±0.7 mV and 45.5±0.6 mV for NC1, *p* = 0.037 and 0.07, respectively). **B and D**, Similar to findings in motor axons, the slope for recovery from hyperpolarization, TE_h(slope 101-140ms)_ is also significantly decreased in sensory axons (1.87±0.13 for patient vs. 2.41±0.06 for NC2, p = 0.002), along with significantly prolonged time to overshoot (49.0±5.0 ms for patients vs. 37.2±1.9 ms for NC2, p = 0.024). Threshold reduction in depolarizing TE in sensory axons is increased in patients (TE_d(10-20ms)_, 75.3±4.1 for patient vs. 60.5±0.8 for NC2, *p* = 0.001). **E and F**, After 200 ms of preconditioning depolarization, there are significantly less threshold reduction in motor axons (47.6±1.4 for carrier vs. 51.6±0.6 for NC1, *p* = 0.014; 47.5±1.9 for patient vs. 51.4±1.1 for NC2, *p* = 0.024). The hyperpolarizing I/V slope is increased in both motor (0.451±0.039 for patient vs. 0.352±0.012 for NC2, p = 0.012) and sensory axons (0.446±0.03 for patient vs. 0.347±0.015 for NC2, p = 0.02) of patients. The lines in parts A to F are all drawn by hand.

To further scrutinize the channel/pump function at nodal and internodal membrane, we investigated threshold electronus (TE) and I/V relationship. In motor axons, there is a smaller percentage of threshold reduction at depolarizing TE in carriers but not in patients (Table 2 and Fig 4A and 4C). On the other hand, the threshold elevation in early hyperpolarizing TE is significantly smaller in patients, (than in NC2, Fig 4C and Table 2). In sensory axons, there is no significant change of the indices of TE curve in carriers. However, the percentage of threshold reduction in depolarizing TE in patients is evidently higher than that of NC2 (Table 2). The patients also show a less steep slope of recovery from hyperpolarization (TE_h(slope 101-140ms)_) and protracted time to overshoot for the patients (Fig 4D and Table 2). This pattern of change is also seen in TE with preconditioning current of 20% of threshold current (Fig 4D). Decrease of the TE_h(slope 101-140ms)_ associated with slowing the time to overshoot indicates a slow recovery from hyperpolarizing TE, which might be due to increase of capacitance and/or increased activities of sodium-potassium pump (See Discussion). The changes of indices in current-threshold (I/V) relationship from patients and carriers are generally insignificant, except that the hyperpolarizing I/V slope in motor axons of patients is higher than that of NC2 (Table 2 and Fig 4E and 4F). Of note, 6 of 18 motor axons and 3 of 7 sensory axons have failed to complete the study due to high threshold after the hyperpolarized preconditioning currents (i.e. -100% of threshold currents).

### Correlation with disease severity

To explore the correlation between the disease severity and the excitability indices, we employed the uni- and multi-variate regression models to analyze the neurological disability score (NDS) and overall neuropathy limitation scale (ONLS). The multivariate regression model shows that age and superexcitability at 5–7 ms are correlated to NDS scores. On the other hand, only age and peak response are clearly correlated to the increase of ONLS scores in ulnar motor axons by univariate regression (Table 3). The correlation between the changes of superexcitability at 5–7 ms and ONLS are only marginally. Since there are more objective assessments in NDS than ONLS, the regression model suggests that superexcitability at relatively short inter-stimulus interval (ie. 5-7ms) is significantly correlated with the disease severity.

**Table 3:** Regression analysis to identify the relationship between the nerve excitability parameters and NDS as well as ONLS.

	Univariate		Multivariate	
Parameters	Beta	P value	Beta	P value
NDS				
Age	0.501	0.034	0.491	0.020
Peak response	-0.437	0.07		
latency	0.434	0.072		
Superexcitability	0.328	0.184		
Superexcitability (5-7ms)	0.475	0.046	0.464	0.027
ONLS				
Peak response	-0.554	0.017	-0.436	0.032
Age	0.585	0.011	0.477	0.021
Superexcitability	0.309	0.212		
Superexcitability (5-7ms)	0.391	0.109		

## Discussion

The study has characterized the changes in motor and sensory axonal membrane excitability in both pre-symptomatic carrier and symptomatic patients with TTR-FAP. We have identified a distinct pattern of membrane dysfunction not only in patients with TTR-FAP but also in carriers before the occurrence of neurological symptoms and discernible abnormalities in conventional nerve conduction studies. TTR-FAP, caused by the p.A97S mutation, is a disease involving both motor and sensory axons from the very early, asymptomatic stage.

### Electrophysiological properties of TTR-FAP

In motor axons, the major findings of NET study in patients and carriers are increased threshold and reobase, decreased threshold reduction in depolarizing TE, and reduced superexcitability. In sensory axons, there are also increase of threshold and rheobase in carriers. These findings strongly indicate a decrease of transient sodium currents on the nodal membrane in both sensory and motor axons in presymptomatic and symptomatic stage of FAP. Another possible contributory or concomitant mechanism is the increase of nodal or internodal slow potassium currents. However, increase of slow potassium currents would increase the subexcitability in the recovery cycle, which is not in our case [24]. Another possible mechanism is the increase of nodal fast potassium current. However, the unchanged resting I/V curve in both carriers and patients would argue against the increase of fast potassium current, which tend to have more significant changes in the resting I/V slope [25]. Interestingly, although the threshold is increased in patients, the extent is apparently not so much as those found in carriers. A possible explanation is that the severely affected axons have degenerated or they could no longer effectively conduct an impulse, especially at the axonal terminals in the relatively late stage of the disease. As compared to carriers, the markedly reduced CMAP in patients provides evidences of axon damage in the late stage. The recovery cycle study for motor axons demonstrates that the refractoriness is higher in patients than those of controls. In younger carriers, the levels of refractoriness and RRP are in the normal range, but there is a trend of gradual increasing by aging. As the disease evolves, both indices escalate to a significantly higher level in the patient group (Fig 3C and 3D). These findings may suggest that the sodium channel dysfunction progresses and aggravates throughout the disease course.

**Fig 5:**
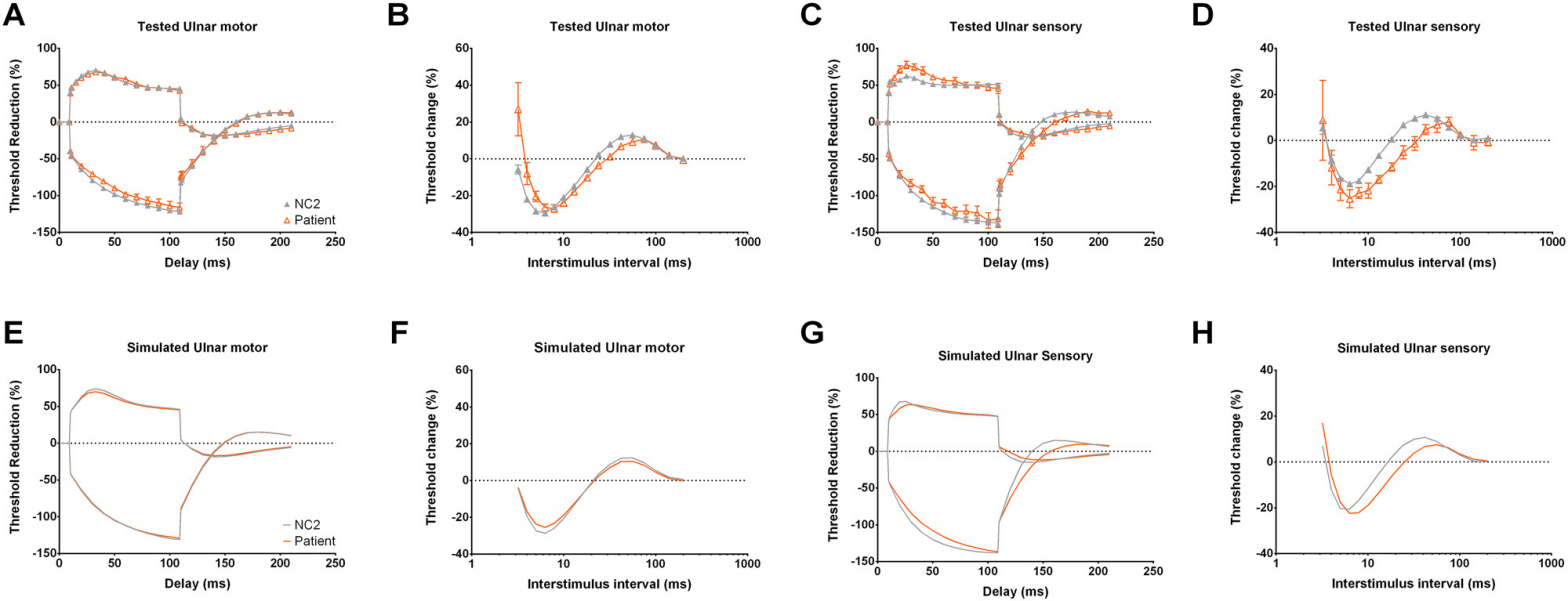
Computer modelling of the properties of nerve excitability (threshold electrotonus and recovery cycle). **A-D**, The results from nerve excitability test of motor and sensory axons from NC2 (the meaning of symbols are the same as in Fig 1) and patients. **E and F**, The gray lines are the simulated excitability curves for NC2, and the orange lines are for patients. For motor axons, the nodal sodium permeability is reduced from 4.1 to 3.75 (cm^-3^ x 10^9^) to simulate the test results shown in **A** and **B**. Based on the motor nerve NET data from patients, we identified three parameters with little discrepancy that are the leak current conductance (1.38 to 2.7 nS), hyperpolarization-activated conductance (6.05 to 7.85 nS) and decrease of nodal transient sodium current permeability (4.1 to 3.75 cm^-3^ x 10^9^). Best fits with changes in these parameters can reduce the discrepancy of 21.7%, 20.9% and 19.8%, respectively. Reduction in sodium currents permeability (4.1 to 3.75 cm^-3^ x 10^9^) causes highest increase of threshold (7.3%), which is the characteristic feature in motor NET findings of patients. In contrast, increase of leak current or hyperpolarization-activated conductance would cause a prominent reduction of threshold elevation after hyperpolarization in I/V curve, which is not found in our patients (Data not shown). **G and H**, For sensory axon simulation, increase of capacitance upon internodal membrane from 0.196 to 0.273 nF is used to simulate the tested results in **C** and **D**. Two parameters in computer models may be changed to fit the results from sensory NET findings from patients. They are the pumping currents and internodal capacitance. With 8.5% reduction of transient sodium channel permeability, increase of the nodal and internodal pumping currents from 11.8 to 21.7 pA and of the internodal capacitance from 0.196 to 0.273 nF could reduce the discrepancy of 68.4% and 38.7%, respectively. The increase in pumping currents, however, is physiologically difficult to envisage in our case (See Discussion). The findings with decrease of the TE_h(slope 101-140ms)_ and protracted the time to overshoot in hyperpolarization TE as shown in patients NET findings (in part **C**) can be replicated in the model with increased internodal capacitance. Similarly, the modeling with increased internodal capacitance also well describes the increase of refractoriness and superexcitability from patients (in part **D)**. The computer simulation demonstrates the likelihood of early changes in nodal sodium conductance and internodal capacitance, but would by no means rule out concomitant minor alterations in the other axonal membrane conductances. The error bars indicate the standard error of mean (S.E.M.). The lines in part-A to -D are drawn by hand.

The NET findings of sensory axons showed prolonged latency to overshoot from hyperpolarizing TE and increased superexcitability in recovery cycle, indicating an increase in capacitance of internodal membrane or increased membrane hyperpolarization in addition to the decreased transient sodium currents. It has been reported that similar changes was observed in patients with chronic demyelinating polyneuropathy and multifocal motor neuropathy with conduction block, which have been considered as the manifestations of impaired myelin integrity, although the indices might be variable in demyelinating neuropathies [26–31]. While adjusting the parameters with increased internodal capacity, the computer simulation model replicates the prolonged time to overshoot in hyperpolarizing TE as well as the increase of superexcitability in the recovery cycle (Fig 5). Alternatively, membrane hyperpolarization could be caused by hyperactive electrogenic sodium-potassium pump which can also demonstrate the fanning-out picture in TE and the increased superexcitability [32]. Hyperactive electrogenic sodium-potassium pump is a common phenomenon during the post-ischemic state [32]. Nevertheless, the membrane hyperpolarization resulting from the increase of sodium-potassium pump activities would decrease the refractoriness in recovery cycle as well as lower the resting I/V slope, both are contrary to our findings. In addition, distal energy failure was shown to be a possible cause of nerve damage in patients with TTR-FAP, [1] which may also preclude the possibility of increasing pumping activity as the main mechanism of pathogenesis.

### Functional insights into the pathological changes in TTR-FAP

Previous studies have suggested that amyloidogenic proteins from mutant TTR can aggregate into oligomers which may interfere with intracellular calcium homeostasis by increasing the permeability of the plasma membrane to extracellular calcium [10, 33]. In the rat dorsal root ganglion neurons, the calcium influxes could be abolished by blockers of voltage-gated calcium channel (VGCC). Application of blockers of voltage-gated sodium channels (eg. Nav1.8) and of transient receptor potential M8 (TRPM8) channel can also decrease the amyloidogenic TTR-induced calcium influxes. Gasperini *et al*. suggested that activation of TRPM8 channels triggers the activation of Nav1.8 channels which in turn leads to calcium influxes through VGCC to contribute to the pathogenesis of FAP [34]. The study addresses that hypofunction or decreased availability of transient sodium conductances might be caused by direct/indirect interactions between mutant TTR and the membrane proteins especially ion channels. On the other hand, Plante-Bordeneuve and Said have demonstrated the disappearance of Schawann cell basal lamina from the patients with FAP on electron microscopy [1]. A recent study with MR neurography also showed increased proton density and prolonged apparent T2 relaxation time in both patients and asymptomatic carriers [35]. These indicators have been used to detect early demyelination changes without significant axonal loss and gliosis in multiple sclerosis [36]. Changes in macromolecular structure and subsequent endoneurial edema might therefore play a major role in the increased internodal capacitance revealed by NET in this study [35]. In a rat sciatic nerve study, topical application of 2-chloroprocaine, an agent acting chiefly by inhibition of the sodium influxes through voltage gated sodium channels on neuronal cell membrane, increased the permeability of perineurium resulting in significant endoneurial edema [37]. Damages of the axonal terminal architecture by deprivation of basal laminae might lead to further decrease in sodium channel density on axonal membrane [38]. These findings underscore the possibility that decreased nodal sodium conductance and increased internodal capacitance in both sensory and motor axons may be interrelated rather than two isolated events.

Amyloid vasculopathy with vessel obliteration and focal demyelination in TTR-FAP patients has been reported in previous pathology studies [39]. However, during ischemia, membrane depolarization caused by dysfunction of sodium-potassium pump would ensue [32]. As we have already mentioned, membrane depolarization is not consistent with the findings in Figs 3B and 4D. Nerve ischemia thus is not evident in our NET findings. A possible explanation is that the obliterated vessel is very small in size and only found in very late stage of polyneuropathy [39]. Also, nerve fibers supplied by those completely obliterated vessels are probably not excitable and consequently not measured in NET. A similar finding has been reported in a NET study in patients with primary amyloidosis [40].

### Clinical and therapeutic implications

The penetrance of TTR FAP is variable from 70% to 90% which means a significant existence of asymptomatic mutant carriers in the affected pedigrees [17, 18]. A few armamentariums are under development such as TTR stabilizers and gene therapy to silence the expression of mutant allele by small interference RNA, antisense oligonucleotides, or specific ribozymes [41–48]. However, these treatments so far can only slow down the progress of the disease, rather than restore the neurologic disability. Orthotopic liver transplantation has been the only curative therapy for TTR-FAP for the past decade [49, 50]. Due to the incomplete penetrance of pathogenic mutations, liver transplantation is not indicated for an asymptomatic carrier. The procedure is also contraindicated in late-stage patients with severe polyneuropathy, severe autonomic dysfunction, or poor nutritional status. Early detection of functional changes is therefore critical for the initiation of the treatment. Several tools, such as superficial sympathetic response and laser evoked potential, have been advocated to evaluate the subjects early in the clinical course [51–53]. Carvalho *et al* in Portugal has reported a nerve excitability study of the TTR-FAP patients in an abstract form [54]. Consistent with our findings, there were increased stimulus for 50% depolarization and rheobase. However, neither significant abnormalities nor data from asymptomatic carriers were described. One of the causes for the discrepancies might also be the difference in TTR mutations i.e. p. A97S in this study but p. V30M in Portugal group. In addition to the mutation genotype, the amyloid polyneuropathy caused by different species of amyloid protein can result in different NET changes [40, 54]. To trace the changes in the NET indices from carriers to patients would be helpful for early detection of clinical penetrance in the carriers (Fig 3C and 3D). More NET studies on different mutant genotypes would be needed to understand whether the pathogenesis would be influenced by the protein-protein interactions among different mutant TTRs. Moreover, it is very likely that NET could serve as an important noninvasive clinical tool to follow up the peripheral nerve function along the protracted course of the disease, i.e. from asymptomatic carriers to symptomatic patients, at least for those carrying specific mutations such as p.A97S.

## Conclusions

In summary, we demonstrated the changes of NET indices in carriers and patients with p.A97S *TTR* mutation. Our results suggest that the pathophysiological hallmarks at the relatively early stages of disease are the decreased availability of transient sodium current at node of Ranvier and focal disruption of basal lamina with increasing of internodal capacity. Loss of axons then emanates to cause permanent neurologic deficit. NET thus could serve as an useful and noninvasive objective tool for early detection and follow-up studies of the functional abnormalities of TTR-FAP.
